# Metastatic Recurrence of Typical Pulmonary Carcinoid Accompanied by Carcinoid Syndrome, Successfully Treated with Octreotide LAR

**DOI:** 10.1155/2017/1564819

**Published:** 2017-12-28

**Authors:** Asako Yanagisawa, Satoshi Hirano, Shinichiro Shimizu, Takuma Hiroishi, Kohei Shikano, Noriko Hayama, Tetsuo Fujita, Hiroyuki Amano, Makoto Nakamura, Sukeyuki Nakamura, Hiroshi Tabeta

**Affiliations:** ^1^Department of Respiratory Medicine, Funabashi Municipal Medical Center, Chiba, Japan; ^2^Department of Medical Oncology, Funabashi Municipal Medical Center, Chiba, Japan; ^3^Department of Pathology, Funabashi Municipal Medical Center, Chiba, Japan

## Abstract

We present a case of metastatic recurrence of carcinoid tumor accompanied by carcinoid syndrome in a 68-year-old Japanese man, 12 years after resection of typical pulmonary carcinoid. Histopathologic examination from percutaneous liver biopsy revealed metastatic typical carcinoid. Clinical symptoms gradually improved after administration of octreotide LAR. Two years after starting treatment, the disease remains well controlled. This case report illustrates the possibility of antiproliferative effects of octreotide LAR on typical pulmonary carcinoid.

## 1. Introduction

Typical pulmonary carcinoid is known as a low-grade subtype of lung neuroendocrine tumor (NET) and is characterized by an indolent nature. Recurrence of typical pulmonary carcinoid after complete resection is very rare (3–5%) [1]. The frequency of carcinoid syndrome among lung NETs is also very low [2]. Somatostatin analogues such as octreotide have been reported to improve the symptoms of carcinoid syndrome [3]. Furthermore, octreotide long-acting repeatable (LAR) has shown antitumor activity against midgut NETs [4].

## 2. Case

**Figure 1:**
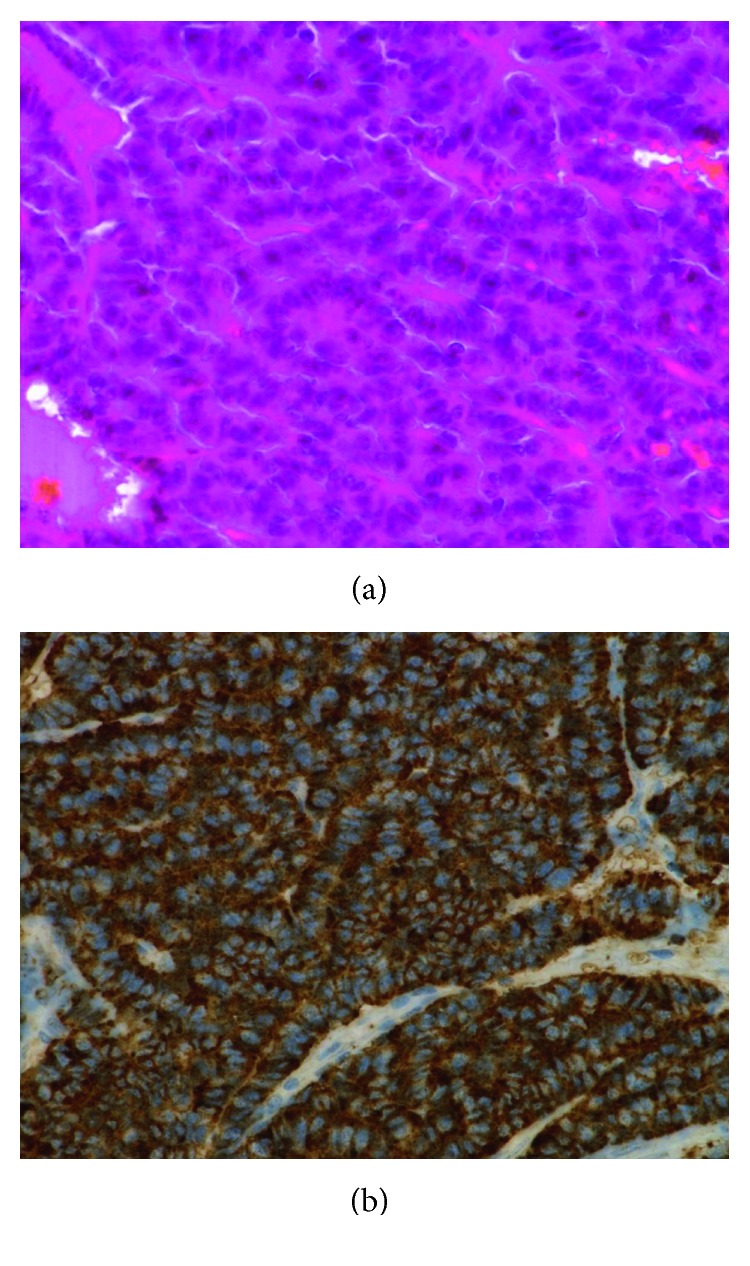
Histologic examination of surgically resected pulmonary carcinoid tumor shows finely granular nuclear chromatin, eosinophilic cytoplasm, and rosette formation (a) and positive expression of chromogranin A (b).

A 68-year-old Japanese man was referred to our hospital because of multiple hyperechoic lesions in the liver without any symptoms. The patient was a former smoker with a 45-pack-year history. Twelve years prior to this presentation, he had undergone thoracoscopic left upper lobectomy for typical pulmonary carcinoid (pT1N0M0, stage I). Histopathologic examination of the surgical specimen revealed finely granular nuclear chromatin, eosinophilic cytoplasm, and rosette formation (Figure 1). The immunohistology was positive for synaptophysin, chromogranin A, and CD56 (Figure 1). The presence of 1 mitosis per 2 mm^2^ and absence of necrosis were consistent with typical carcinoid. The hilar and mediastinal lymph nodes were negative for any tumor, and the surgical margins as well were free of any tumor.

**Figure 2:**
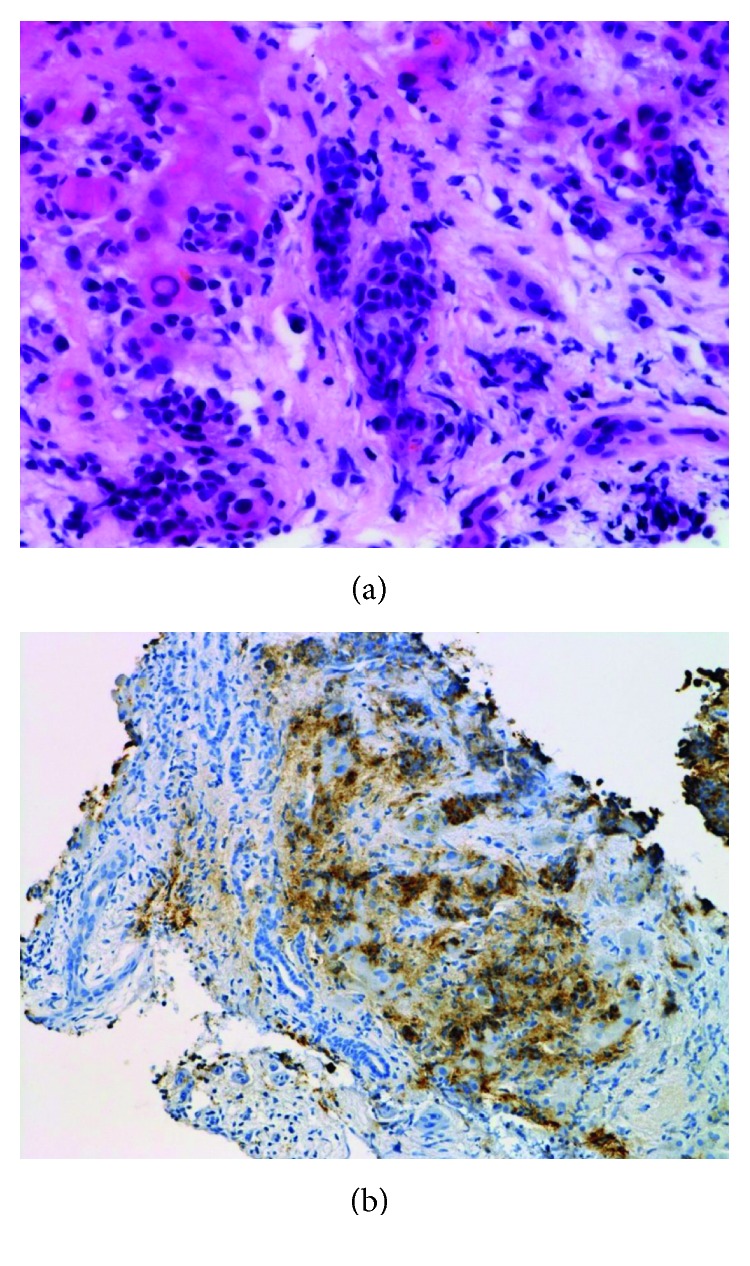
Histologic examination of a liver biopsy specimen shows a well-differentiated tumor (a) with positive expression of chromogranin A (b).

The patient had a history of atrial fibrillation. Physical examination revealed no abnormalities. Computed tomography (CT) with intravenous contrast of the abdomen revealed multiple low-density areas. Percutaneous liver biopsy of one of the lesions demonstrated well-differentiated tumor with positive expressions of chromogranin A, synaptophysin, and CD56 (Figure 2). The presence of 1 mitosis per 2 mm^2^ and no necrosis were also demonstrated, which was compatible for metastatic typical carcinoid. During 6 months of close observation, the patient developed chronic diarrhea resulting in 12 kg of weight loss. His general condition worsened, and he was hospitalized.

**Figure 3:**
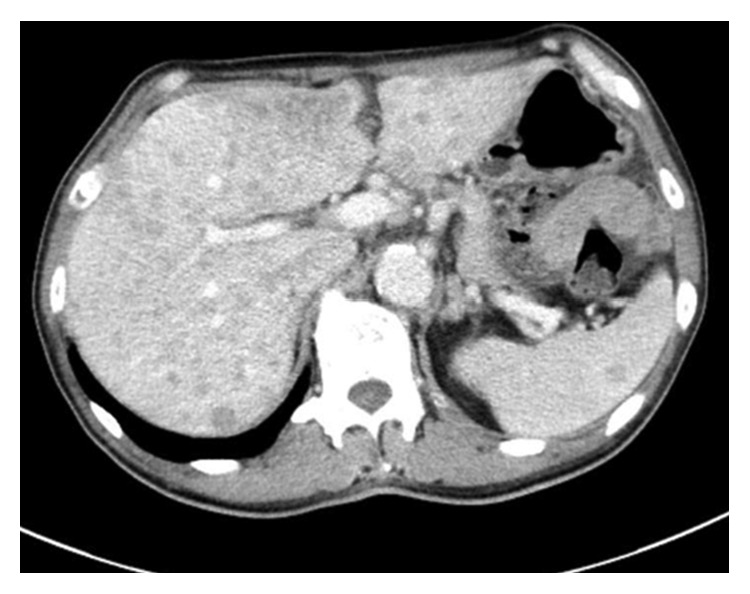
Abdominal contrast-enhanced CT shows multiple low-density areas, hepatomegaly, and metastasis to the spleen.

Physical examination showed hepatomegaly and wheezing. Levels of neuron-specific enolase and *α*-fetoprotein were both elevated, at 24.7 ng/ml (normal range (NR) < 16.3 ng/ml) and 56 ng/ml (NR < 10 ng/ml), respectively. Blood 5-hydroxyindole acetate (5-HIAA), urine 5-HIAA, and serotonin degradation metabolite levels were elevated at 1121 ng/ml (NR 57–230 ng/ml), 472 ng/ml (NR 1.8–6.1 ng/ml), and 86.9 mg/13 h (NR 1–6 mg/24 h), respectively, confirming the diagnosis of carcinoid syndrome. Chest CT showed no abnormalities. Abdominal contrast-enhanced CT revealed multiple tumors in the liver and spleen, as well as hepatomegaly (Figure 3). Magnetic resonance imaging (MRI) of the brain revealed an enhanced nodular lesion in the right occipital lobe, compatible with a brain metastasis.

**Figure 4:**
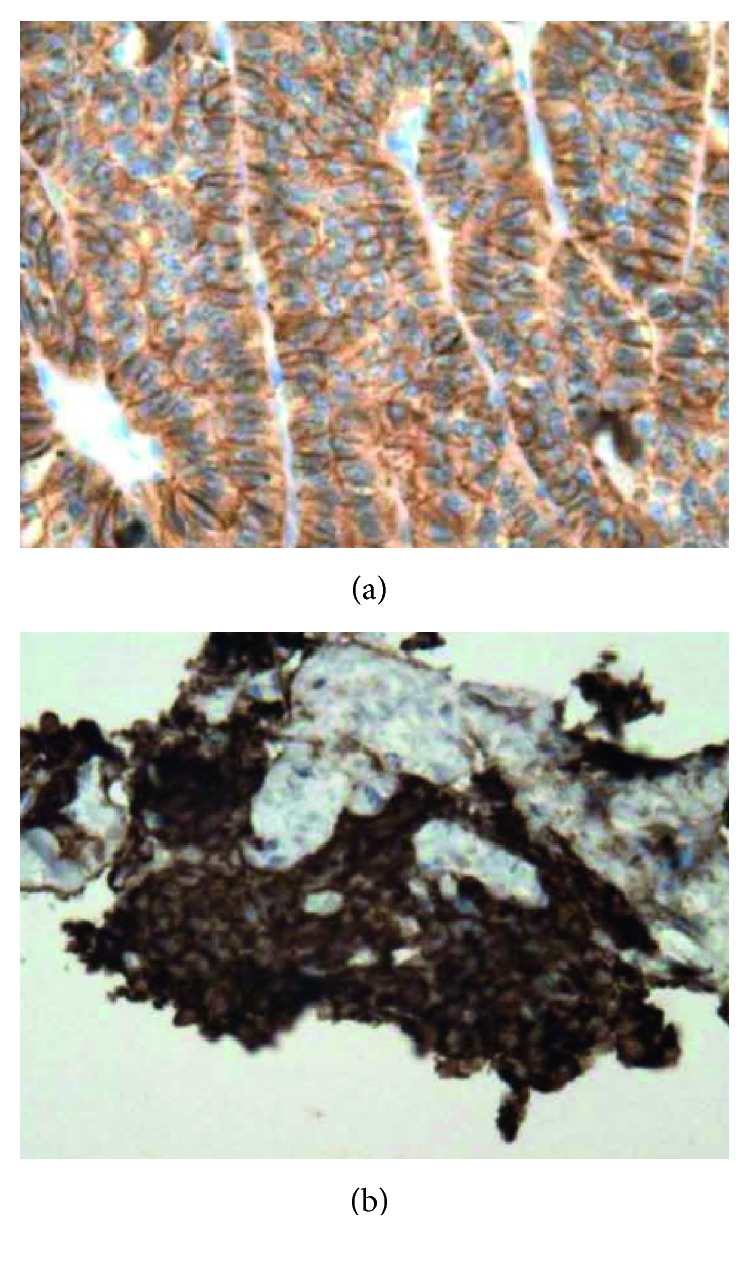
Immunohistochemical analysis demonstrates immunoreactivity for somatostatin receptor 2 antibody immunostaining in most tumor cells in the lung (a) and liver (b).

Considering that immunostaining for somatostatin receptor 2 antibody was scored as 3 (circumferential membranous reactivity in >50% of tumor cells), subcutaneous octreotide therapy was started at a dose of 100 *μ*g/day (Figure 4). Blood serotonin, blood 5-HIAA, and neuron-specific enolase levels decreased markedly and diarrhea gradually improved. Following 20 mg of LAR octreotide via intramuscular injections every 4 weeks, the dose was increased to 30 mg every 4 weeks because of persistent diarrhea. Two years after starting octreotide therapy, the disease is well controlled with almost no symptoms, except for transient ischemic attack.

## 3. Discussion

Pulmonary carcinoid tumor is a rare NET that accounts for less than 2% of all lung tumors. Good prognosis can be expected following complete surgical resection of pulmonary carcinoid tumor. The recurrence rate for typical carcinoid is lower (3.6%) than that of atypical carcinoid (33.3%) [5]. Histological subtype, staging, and nodal status are reported to be significant predictors of disease-free survival [6]. In contrast, Maurizi showed that only histological subtype (and not nodal status) can influence disease-free survival, with a statistically significant advantage for typical carcinoid [7]. The distinction between typical and atypical carcinoid is thus very important in the determination of prognosis. The WHO 2015 classification for lung NETs states that the defining features of atypical carcinoid are the presence of 2–10 mitoses per 2 mm^2^ and/or the presence of necrosis [8]. As for atypical carcinoid, Arrigoni et al. [9] first described the histological criteria for atypical bronchopulmonary carcinoid tumors in 1972, namely, increased mitotic activity with 5–10 mitoses per 2 mm^2^ (10 high-power field (HPF)), nuclear pleomorphism, and irregularity with hyperchromatism and prominent nucleoli, areas of increased cellularity with disorganization, and necrosis. Travis et al. reported increased mitotic activity as the only independent predictor of prognosis in the criteria described by Arrigoni [10]. He proposed that either a mitotic count ≥ 2 and <10 per 2 mm^2^ (10 HPF) in viable tumor or the presence of necrosis should be the histologic criterion for atypical carcinoid, because atypical carcinoid would be underestimated if the mitotic range is set between 5 and 10 mitoses (10 high-powered fields), and the presence of necrosis is indicative of poor prognosis.

Only a small number of patients with typical carcinoid experience recurrences, with a median time to recurrence of 4 years (range, 0.8–12 years), longer than that for atypical carcinoid [11]. As a result, few case reports have addressed the late recurrence of typical carcinoid tumors. Our case was valuable in that liver metastases occurred more than 10 years after complete resection of the primary tumor.

The primary lesion was diagnosed as typical carcinoid. However, part of the primary lesion showed conspicuous nucleoli and high cellularity, consistent with atypical carcinoid according to the former criteria [9]. These features may indicate the potential for recurrence or metastasis of typical carcinoid. Arrigoni et al. reported that 70% of 23 carcinoids with such histologic features exhibited metastasis, as compared to 5.6% of histologically typical carcinoid [9]. Atypical features including high cellularity and prominent nucleoli could be assumed to be related to metastasis to the liver, and the low mitotic count was associated with late recurrence in our case.

Whether liver metastases originated from the lung in our case represents an important issue, because there is a possibility of the presence of another primary site. When liver metastases are demonstrated, CT of the chest and abdomen, abdominal ultrasonography, upper gastrointestinal endoscopy, and colonoscopy are performed to exclude primary tumors other than those of the lung, but no other gastroenteropancreatic neuroendocrine tumors were found in our case. Brain metastasis without pulmonary lesions is not common for primary sites other than the lung.

Patients with carcinoid syndrome display a shorter overall survival (median, 5 years) than patients without carcinoid syndrome (5.6 years) [2]. Patients with NETs and carcinoid syndrome show marked impairments in multiple areas. Carcinoid syndrome also affects patients' quality of life. Although the frequency of carcinoid syndrome in lung NETs is very low, our patient was diagnosed with typical pulmonary carcinoid and carcinoid syndrome.

Somatostatin receptor scintigraphy (SRS) is useful for the diagnosis of neuroendocrine tumors [12]. Accumulations on SRS reflect the degree of tumor differentiation, and SRS shows high sensitivity for the detection of low-grade neuroendocrine tumors. SRS is a helpful option for detecting metastatic lesions that are hard to find by other imaging modalities and for predicting the outcomes of octreotide treatment [13]. Although we did not perform SRS, strongly positive results for somatostatin receptor 2 were seen on immunohistochemical staining [14]. Octreotide suppresses the production of physiologically active substances through binding to somatostatin receptors on tumor cells [15]. Octreotide can be expected to have somatostatin receptor-mediated and antiangiogenic antiproliferative effects [16].

The LAR formulation of octreotide inhibits tumor growth in patients with metastatic midgut neuroendocrine tumors [4]. Octreotide treatment resulted in control of carcinoid syndrome and tumor reduction or complete regression in 43% of patients with liver metastases of pulmonary atypical carcinoid [17]. An antiproliferative effect of octreotide on pulmonary carcinoid tumor can be expected. However, few case reports have addressed the antiproliferative effects of octreotide LAR on typical pulmonary carcinoid. An antiproliferative effect of another long-acting somatostatin analogue lanreotide was demonstrated in a study of more than 200 patients with somatostatin receptor-positive enteropancreatic neuroendocrine tumors with Ki-67 values of less than 10% [18]. Although the patients in this study were thought to have more indolent tumors, the estimated rates of progression-free survival were 65.1% in the lanreotide group and 33.0% in the placebo group. In our case, carcinoid syndrome was well controlled after administration of octreotide LAR, which also resulted in a long period of disease stabilization. Considering the median PFS of 11.3 months in the placebo plus octreotide LAR group in the RADIANT-2 trial [3], our case may indicate that treatment with octreotide LAR is associated with longer tumor stabilization in typical carcinoid, which has the lower mitotic rate.

In summary, this case report illustrates the possibility of antiproliferative effects of octreotide LAR on typical pulmonary carcinoid.
